# Exposure to volatile organic compounds and chronic respiratory disease mortality, a case-cohort study

**DOI:** 10.1186/s12931-025-03165-1

**Published:** 2025-03-05

**Authors:** Mahdi Nalini, Hossein Poustchi, Deepak Bhandari, Benjamin C. Blount, Brandon M. Kenwood, Cindy M. Chang, Amy Gross, Christopher Ellison, Masoud Khoshnia, Akram Pourshams, Mitchell H. Gail, Barry I. Graubard, Sanford M Dawsey, Farin Kamangar, Paolo Boffetta, Paul Brennan, Christian C. Abnet, Reza Malekzadeh, Neal D. Freedman, Arash Etemadi

**Affiliations:** 1 Metabolic Epidemiology Branch, Division of Cancer Epidemiology and Genetics, National Cancer Institute, National Institutes of Health, Bethesda, MD USA; 2 Digestive Oncology Research Center, Digestive Diseases Research Institute, Tehran University of Medical Sciences, Tehran, Iran; 3 Liver and Pancreaticobilliary Research Center, Digestive Diseases Research Institute, Tehran University of Medical Sciences, Tehran, Iran; 4 Division of Laboratory Sciences, National Center for Environmental Health, Centers for Disease Control and Prevention, Atlanta, GA USA; 5 Center for Tobacco Products, Food and Drug Administration, Silver Spring, MD USA; 6 Biostatistics Branch, Division of Cancer Epidemiology and Genetics, National Cancer Institute, National Institutes of Health, Bethesda, MD USA; 7 Department of Biology, School of Computer, Mathematical, and Natural Sciences, Morgan State University, Baltimore, MD USA; 8 Stony Brook Cancer Center, Stony Brook University, Stony Brook, NY USA; 9 Department of Medical and Surgical Sciences, University of Bologna, Bologna, Italy; 10 International Agency for Research on Cancer, Lyon, France

**Keywords:** Air pollution, Pulmonary disease, Asthma, Tobacco smoking

## Abstract

**Background:**

Chronic respiratory diseases (CRDs) are the third leading cause of death worldwide. Data of the associations between specific volatile organic compounds (VOCs), a major component of air pollution and tobacco smoke, and subsequent CRD mortality in the general population are scarce.

**Methods:**

In a case-cohort analysis within the population-based Golestan cohort study (*n* = 50045, aged 40–75 years, 58% women, enrollment: 2004–2008, northeastern Iran), we included all participants who died from CRD during follow-up through 2018 (*n* = 242) as cases and stratified them into 16 strata defined by age, sex, residence, and tobacco smoking. Subcohort participants (*n* = 610) were randomly selected from all eligible cohort participants in each stratum, and sampling fractions were calculated. Baseline urine samples were used to measure 20 VOCs using ultra high-performance liquid chromatography coupled with electrospray ionization tandem mass spectrometry. After excluding participants with previous history of CRDs, we used stratified Cox regression models weighted by the inverse sampling fractions (i.e. inverse probability weighting) adjusted for potential confounders, including urinary cotinine and pack-years of smoking, to calculate hazard ratios (HR) for the associations between biomarker tertiles and CRD mortality.

**Results:**

Data from 545 non-case, sub-cohort participants and 149 cases (69.1% chronic obstructive pulmonary disease, 13.4% asthma, 17.5% other CRDs) were assessed in this study. During a follow-up of 10.5 years, associations [2nd and 3rd vs. 1st tertiles, HR (95% confidence interval), p for trend] were observed between metabolites of acrolein [1.56 (0.64,3.79), 3.53 (1.53,8.16), 0.002] and styrene/ethylbenzene [1.17 (0.53,2.60), 3.24 (1.37,7.66), 0.005] and CRD mortality, which persisted after excluding the first four years of follow-up.

**Conclusion:**

Our findings support prior research suggesting respiratory toxicity of VOCs. Further investigation and monitoring of these compounds, especially acrolein and styrene/ethylbenzene, as CRD risk factors, are recommended.

**Supplementary Information:**

The online version contains supplementary material available at 10.1186/s12931-025-03165-1.

## Introduction

Chronic respiratory diseases (CRDs) are among the main causes of premature mortality and morbidity worldwide [1]. With 4.0 million deaths and 455 million prevalent cases in 2019, they constituted the third leading cause of death globally [2]. While chronic obstructive pulmonary disease is the main contributor to mortality from CRDs, asthma has the highest prevalence [2]. However, CRDs have received little attention from healthcare providers and policymakers compared with other noncommunicable diseases such as cardiovascular diseases and cancers [3, 4]. This is while a substantial proportion of the disease burden is preventable by eliminating exposure to tobacco smoke and air pollution [1, 2]. 

Complex mixtures of volatile organic compounds (VOCs) comprise one of the major groups of toxic chemicals in tobacco smoke. They also stem from a myriad of human-produced sources such as fossil fuels, solvents, adhesives, cleaning agents, pesticides, cosmetics, and cooking fumes [5, 6]. Because of their high volatility and broad production, VOCs are widely present in the environment and can potentially cause health effects, especially in the respiratory system as the main route of exposure [6, 7]. 

Detection of individual VOCs associated with CRDs is critical for biomonitoring and prevention of diseases, however, such studies are limited because of several constraints. Epidemiological studies on the respiratory effects of VOCs have mainly focused on specific occupational settings with high levels of VOCs and limited generalizability [8, 9]. Another limitation is measurements of exposure and outcome at the same time in case-control or cross-sectional designs, without adequate consideration of exposure-outcome temporality [8, 10, 11]. For example, some respiratory diseases may influence the respiratory absorption of certain toxicants, potentially introducing bias into exposure measures. Longitudinal studies that measure exposure to VOCs at baseline, ideally at multiple time points, and subsequently assess outcomes during follow-up would be essential to evaluate the temporality of this relationship [8, 10]. A common method of exposure assessment is the measurement or estimation of environmental VOCs, while the measurement of the internal dose through biomarkers is the preferred approach since it considers all types of VOC sources as well as individual variations in absorption and metabolism [11]. Urinary mercapturic acid metabolites of VOCs are standard biomarkers for biomonitoring of VOC exposure and provide accurate data because of the relatively long physiological half-lives and high specificity [6]. Nevertheless, few prospective cohort studies have examined the association of VOC exposure with subsequent respiratory disease incidence, progression, or death; as only a limited number of cohort studies have urine samples available for such analyses [12]. 

Here we evaluate the associations of 20 urinary VOC biomarkers **(**Table 1**)** with CRD mortality in the Golestan Cohort Study (GCS). The GCS is one of the few prospective cohorts with long follow-up and baseline urine samples available from the general population. We conducted this study using a collaboration between the U.S. Food and Drug Administration, Center for Tobacco Products, the Division of Laboratory Sciences of the National Center for Environmental Health at the Centers for Disease Control and Prevention (CDC), and the Division of Cancer Epidemiology and Genetics at the National Cancer Institute.

**Table 1:** List of urinary biomarkers and their parent volatile organic compounds

Full name of biomarker	Abbreviations	Parent compound
2-Methylhippuric acid	2MHA	o-Xylene
3- and 4-Methylhippuric acid	3&4MHA	m- and p-Xylene
2-Carbamoylethyl mercapturic acid	2CaEMA	Acrylamide
2-Carbamoyl-2-hydroxy-ethyl mercapturic acid	2CaHEMA	Acrylamide
1-Cyano-2-hydroxyethyl mercapturic acid	1CyHEMA	Acrylonitrile
2-Cyanoethyl mercapturic acid	2CyEMA	Acrylonitrile
2-Hydroxyethyl mercapturic acid	2HEMA	1,2-Dibromoethane; Vinyl chloride; Ethylene oxide, Acrylonitrile
2-Carboxyethyl mercapturic acid	2CoEMA	Acrolein
3-Hydroxypropyl mercapturic acid	3HPMA	Acrolein
Benzyl mercapturic acid	BzMA	Toluene; Benzyl alcohol
Mandelic acid	MADA	Styrene, ethylbenzene
Phenylglyoxylic acid	PhGA	Styrene, ethylbenzene
Phenyl mercapturic acid	PhMA	Benzene
2-Hydroxypropyl mercapturic acid	2HPMA	Propylene oxide
Methylcarbamoyl mercapturic acid	MCaMA	N, N-Dimethylformamide; Methyl-isocyanate
3,4-Dihydroxybutyl mercapturic acid	34HBMA	1,3 Butadiene
4-Hydroxy-2-buten-1-yl mercapturic acid	4HBeMA	1,3 Butadiene
3-Hydroxy-1-methylpropyl mercapturic acid	3HMPMA	Crotonaldehyde
4-Hydroxy-2-methyl-2-buten-1-yl mercapturic acid	4HMBeMA	Isoprene
2-Thioxothiazolidine-4-carboxylic acid	TTCA	Carbon-disulfide

## Methods

### Study design

This study is a case-cohort analysis within the population-based GCS. The GCS enrolled 50,045 participants, 58% women, aged 40–75 years, between 2004 and 2008, from Gonbad City (*n* = 10,032) and 326 villages (*n* = 40,013) in Golestan Province, northeastern Iran. The study was designed to investigate risk factors for chronic diseases, with an emphasis on esophageal cancer because of its high incidence in the study area. The participation rate of cohort was 73.2% (80.6% women and 65.2% men). Details of the GCS have been described previously [13]. For this case-cohort analysis, the eligible cases were all participants who died from CRDs up until January 1, 2018 (*n* = 242) who were stratified into 16 strata defined by age (< 55, ≥ 55 years), sex (male, female), place of residence (rural, urban), and current smoking status (yes, no). As the reference sub-cohort, we selected random samples of 610 individuals in the same 16 strata, from among all cohort participants with available urine samples (*n* = 47,155), regardless of their outcome during the follow-up (TableS1). Table S2 shows baseline characteristics of the subcohort and all cohort participants.

### Urinary biomarker assays

A spot urine sample was collected from all participants at the baseline cohort. Twenty VOC biomarkers were measured by the Division of Laboratory Sciences of the National Center for Environmental Health (NCEH) at the CDC, Atlanta, GA (Table 1), using ultra high-performance liquid chromatography coupled with electrospray ionization tandem mass spectrometry [6]. The limit of detection (LOD) for these VOC biomarkers ranged from 0.5 to 13 ng/mL (Table S3). We included masked vials containing pooled quality control samples randomly placed in the batches sent to the laboratory, and we used these samples to calculate assay coefficients of variation (CV). All the assays had CVs below 15%, except for the 4HMBeMA biomarker which had a CV of 17% (Table S3). We also measured urinary cotinine concentrations using a modified version of isotope dilution high-performance liquid chromatography/atmospheric pressure chemical ionization tandem mass spectrometry [14]. The LOD for cotinine was 0.03 ng/mL. Urine creatinine was measured using a commercial automated, colorimetric enzymatic (creatinase) method implemented on a Roche/Hitachi cobas 6000 Analyzer.

### Covariate assessment

Demographics and medical history data were collected by trained interviewers at enrollment. Detailed data of tobacco use were collected. Tobacco use was defined as active use of tobacco at least once a week for six months. Former tobacco users included those who quit more than one year before cohort enrolment. The amount of smoking (pack-year) was calculated by multiplying cigarette packs (20 cigarettes in a pack) per day by years of smoking. Data of nass (the local chewing tobacco) consumption was also recorded. There was excellent agreement between high urinary cotinine concentration and self-reported current tobacco use (kappa = 0.91 ± 0.08) [15]. The history of previous CRDs were recorded using this question: “has a doctor ever diagnosed you with a chronic obstructive pulmonary disease (asthma/chronic bronchitis/emphysema)?” A wealth score was calculated from appliance ownership, using multiple correspondence analysis [16]. Physical activity was calculated based on metabolic equivalents (METs.min/week). The body-mass index (BMI, kg/m^2^) was calculated using standard measured weight and height.

### Follow-up and outcome ascertainment

All participants are followed annually by phone calls or home visits. In the case of death, two internists determine the cause of death independently based on all relevant medical documents and the result of verbal autopsy, if necessary [17]. In case of disagreement, a third senior internist reviews all documents and the two initial diagnoses to make the final diagnosis. For the present study, causes of death other than CRDs were censored at date of death. Those who were still alive at the end of follow-up (January 1, 2018) or were lost to follow-up for other reasons were censored at the date of their last follow-up. We considered CRD mortality based on the International Classification of Diseases (ICD-10), J codes, including chronic obstructive pulmonary diseases (J40-J44) and asthma (J45).

### Statistical methods

Participants with urine creatinine values outside the plausible range of 10–370 mg/dL or technical problems in urinary biomarker assays were excluded (*n* = 14). We also excluded participants with a history of CRD at baseline (*n* = 140), as baseline CRDs can reduce VOC absorption through the lung and lead to biased estimates (Table S4). Finally, the data from 545 non-case, sub-cohort participants and 149 cases were included in this study.

Biomarkers with below the LOD concentrations were replaced using multiple imputation via interval regression to keep the imputed values below the LOD. The biomarker concentrations were divided by urinary creatinine to adjust for urinary dilution (direct creatinine correction). Geometric means (GM) and corresponding 95% confidence intervals (95% CI) of creatinine-corrected biomarkers were calculated, and their natural logarithms were compared using independent t tests. Using stratified Cox proportional hazards models, based on the 16 strata of cases and non-cases (Table S1), we calculated hazard ratios (95% CIs) for the association between VOC biomarkers (tertiles of the creatinine-corrected biomarkers) with CRD death, according to the Barlow method for analyzing the case-cohort design [18]. For these analyses, the participants in all strata were weighted by the inverse of the sampling fractions (inverse probability weighting). We used age as the time metric in this study. By using stratified Cox regression, individuals in each stratum are compared with similar individuals with regards to age group, sex, place of residence and current smoking. We excluded biomarkers with more than 50% below the LOD concentrations (2CaHEMA, 1CyHEMA, and TTCA) from these analyses. The Cox models were adjusted for age (continuous), ethnicity (Turkmen/non-Turkmen), marital status (married/non-married), years of schooling (0, ≤ 5, >5 years), BMI (< 18.5, 18.5–24.9, 25-29.9, ≥ 30 kg/m^2^) physical activity (tertiles), wealth score (tertiles), nass use (never/ past/current), creatinine-corrected cotinine, pack-years of smoking, and history of cardiovascular events (myocardial infarction or stroke). The underlying covariates were determined a priori, based on a comprehensive literature review. We calculated P-values for trends using medians of biomarker concentrations in each tertile and used multiple comparisons correction with Benjamini and Hochberg’s method to false discovery rate control [19]. Multiplicative interactions were evaluated between the tertiles of biomarkers and smoking status, by including interaction terms in the regression models. The proportionality of hazards was verified using Schoenfeld’s global test. In a sensitivity analysis, we used non-creatinine-corrected biomarker concentrations and included creatinine as a covariate in the models to re-evaluate our findings with another common method of urinary biomarker studies as opposed to direct creatinine correction. In another sensitivity analysis, we excluded the first four years of follow-up (i.e., *n* = 63 individuals who died from any cause in the first four years of follow-up including 37 cases who died from CRDs) to decrease the reverse causality bias due to the long-term pattern of CRD development. There were no missing values for VOC biomarkers in our study, except for 2CaHEMA and MADA in one urine sample. Physical activity levels were missing in 13 cases and 23 non-cases, so we used separate missing indicators to keep them in the models. All statistical analyses were conducted with the Stata statistical software, version 17 (StataCorp, College Station, TX).

## Results

The participants had a total of 6704 person-years of follow-up, with a median (25th − 75th percentiles) of 10.5 (8.1–11.2) years. The mean (SD) age at baseline was 58.0 (9.5) years and 251 (36.2%) were women. The mean (SD) of age at death was 67.3 (10.2) in CRD cases. Among 149 cases who died from respiratory disease, 103 (69.1%) died from chronic obstructive pulmonary disease, 20 (13.4%) from asthma, and 26 (17.4%) from others/unspecified respiratory diseases. The cases compared with non-cases were more likely to have a low BMI, less formal schooling, lower wealth scores, and less physical activity (Table 2**)**. In both cases and non-cases, individuals in the smoking subgroups were more likely to have a lower BMI than participants of the non-smoking subgroups. Medians (25th – 75th percentiles) of pack-years of cigarette smoking were 23.0 (15.0-42.5) and 16.0 (4.3–31.0), respectively, in cases and non-cases who reported cigarette smoking.

**Table 2:** Baseline characteristics of the cases and non-cases by smoking subgroups

Variables		Cases			Non-cases		
		All (*n* = 149)	Smoking (*n* = 45)	Non-smoking (*n* = 104)	All (*n* = 545)	Smoking (*n* = 185)	Non-smoking (*n* = 360)
Age (years)		60.41 (9.40)	60.51 (7.71)	60.36 (10.08)	57.38 (9.46)	56.60 (9.80)	57.79 (9.27)
Sex, women		52 (34.90)	2 (4.44)	50 (48.08)	199 (36.51)	19 (10.27)	180 (50.00)
Residence, rural		117 (78.52)	31 (68.89)	86 (82.69)	404 (74.13)	133 (71.89)	271 (75.28)
Ethnicity, Turkmen		105 (70.47)	36 (80.00)	69 (66.35)	377 (69.17)	133 (71.89)	244 (67.78)
Marital status, married		120 (80.54)	41 (91.11)	79 (75.96)	469 (86.06)	172 (92.97)	297 (82.50)
Previous cardiovascular events		18 (12.08)	3 (6.67)	15 (14.42)	44 (8.07)	17 (9.19)	27 (7.50)
Body-mass index (kg/m^2^)	< 18.5	41 (27.52)	19 (42.22)	22 (21.15)	49 (8.99)	30 (16.22)	19 (5.28)
	18.5–24.9	75 (50.34)	23 (51.11)	52 (50.00)	235 (43.12)	92 (49.73)	143 (39.72)
	25-29.9	14 (9.40)	3 (6.67)	11 (10.58)	160 (29.36)	44 (23.78)	116 (32.22)
	≥ 30	19 (12.75)	0	19 (18.27)	101 (18.53)	19 (10.27)	82 (22.78)
Education	No	118 (79.19)	30 (66.67)	88 (84.62)	374 (68.62)	109 (58.92)	265 (73.61)
	≤ 5 years	18 (12.08)	8 (17.78)	10 (9.62)	92 (16.88)	39 (21.08)	53 (14.72)
	> 5 years	13 (8.72)	7 (15.56)	6 (5.77)	79 (14.50)	37 (20.00)	42 (11.67)
Wealth score	1st terile	72 (48.32)	25 (55.56)	47 (45.19)	223 (40.92)	74 (40.00)	149 (41.39)
	2nd tertile	48 (32.21)	9 (20.00)	39 (37.50)	146 (26.79)	51 (27.57)	95 (26.39)
	3rd tertile	29 (19.46)	11 (24.44)	18 (17.31)	176 (32.29)	60 (32.43)	116 (32.22)
Physical activity	1st terile	78 (52.35)	23 (51.11)	55 (52.88)	183 (33.58)	45 (24.32)	138 (38.33)
	2nd tertile	23 (15.44)	4 (8.89)	19 (18.27)	154 (28.26)	50 (27.03)	104 (28.89)
	3rd tertile	35 (23.49)	15 (33.33)	20 (19.23)	185 (32.94)	82 (44.32)	103 (28.61)
	unknown	13 (8.72)	3 (6.67)	10 (9.62)	23 (4.22)	8 (4.32)	15 (4.17)
Nass use	Never users	111 (74.50)	36 (80.00)	75 (72.12)	483 (88.62)	160 (86.49)	323 (89.72)
	Past users	9 (6.04)	1 (2.22)	8 (7.69)	15 (2.75)	5 (2.70)	10 (2.78)
	Current users	29 (19.46)	8 (17.78)	21 (20.19)	47 (8.62)	20 (10.81)	27 (7.50)

Data are mean (SD) or n (%)

Table 3 shows the GMs of biomarkers in cases and non-cases. The concentrations of 2CyEMA, 2CoEMA, MADA, and MCaMA were higher in cases who died from CRDs compared to non-cases. The GMs of nearly all biomarkers in participants who smoked tobacco were higher than those who did not smoke (Table S5). Table S6 presents the correlations between creatinine-corrected VOC biomarkers in the subcohort. Most biomarkers derived from a common parent compound were highly correlated (e.g., acrylamide, acrylonitrile, and acrolein metabolites, with Pearson coefficients ranging from 0.75 to 0.93).

**Table 3:** Geometric means and 95% confidence intervals of volatile organic compound biomarkers (µg/g creatinine)

	Cases (*n* = 149)	Non-cases (*n* = 545)
2MHA	112.48(90.70,139.49)	109.26(99.05,120.52)
3&4MHA	499.88(407.61,613.04)	446.69(404.77,492.96)
2CaEMA	79.94(65.85,97.04)	76.35(70.48,82.72)
2CaHEMA	9.30(7.58,11.41)	8.86(7.98,9.84)
1CyHEMA	4.03(2.91,5.59)	3.35(2.87,3.91)
2CyEMA	9.50(6.48,13.93)	6.14(5.03,7.49)*
2HEMA	1.88(1.54,2.29)	1.92(1.73,2.13)
2CoEMA	134.20(115.96,155.31)	111.12(103.65,119.13)*
3HPMA	391.88(314.55,488.23)	344.43(314.30,377.44)
BzMA	6.50(5.43,7.78)	5.68(5.27,6.13)
MADA	335.14(298.69,376.03)	258.65(244.60,273.50)*
PhGA	77.48(61.66,97.37)	95.13(86.43,104.69)
PhMA	1.09(0.91,1.30)	1.03(0.94,1.12)
2HPMA	30.04(25.58,35.29)	31.40(29.06,33.92)
MCaMA	230.76(193.59,275.07)	178.84(163.54,195.57)*
34HBMA	351.15(312.46,394.63)	338.54(322.27,355.63)
4HBeMA	9.52(7.51,12.06)	8.94(8.13,9.83)
3HMPMA	377.51(307.52,463.42)	350.47(319.80,384.08)
4HMBeMA	6.29(4.76,8.31)	5.62(4.96,6.37)
TTCA	8.01(6.40,10.03)	6.90(6.09,7.82)

*p < 0.05. Abbreviations are defined in Table 1

Table 4 shows associations between VOC biomarker concentrations and CRD mortality in adjusted models. There was a significant association between the highest tertile (vs. the lowest tertile) of acrolein biomarker 2CoEMA, (HR = 3.53; 95%CI: 1.53,8.16, p for trend = 0.0017) and CRD mortality. The styrene/ethylbenzene biomarker MADA was also significantly associated with CRD mortality (HR 3rd vs. 1st tertile = 3.24; 95%CI: 1.37,7.66, p for trend = 0.0045). Including interaction terms between tertiles of these biomarkers and smoking status did not change the results. Table 5 shows that in both sensitivity analyses, the associations of these acrolein and styrene/ethylbenzene biomarkers with CRD mortality remained significant after using creatinine adjustment in the model instead of direct creatinine correction, and after excluding the first four years of follow-up. When we used creatinine adjustment in the model, as opposed to direct creatinine correction, the highest tertile of another acrolein biomarker, 3HPMA, showed a significant association with CRD death (HR = 3.58; 95%CI: 1.12,11.50).

**Table 4:** Associations of volatile organic compound biomarker tertiles and chronic respiratory disease mortality in the Golestan cohort study

	2nd vs. 1^St^ tertile	3rd vs. 1st tertile	*P* for trend	Corrected reference *P* values*
2MHA	1.10(0.56,2.17)	1.44(0.70,2.96)	0.3020	0.0265
3&4MHA	1.20(0.53,2.74)	1.49(0.71,3.14)	0.3000	0.0235
2CaEMA	0.85(0.40,1.79)	0.75(0.30,1.90)	0.6072	0.0441
2CyEMA	1.02(0.49,2.11)	1.43(0.48,4.28)	0.4726	0.0324
2HEMA	0.34(0.15,0.76)	0.60(0.27,1.32)	0.6783	0.0471
2CoEMA	1.56(0.64,3.79)	3.53(1.53,8.16)	0.0017†	0.0029
3HPMA	0.83(0.38,1.83)	3.08(0.92,10.35)	0.0444	0.0088
BzMA	0.72(0.35,1.47)	1.11(0.54,2.28)	0.5238	0.0353
MADA	1.17(0.53,2.60)	3.24(1.37,7.66)	0.0045†	0.0059
PhGA	0.42(0.19,0.93)	0.64(0.33,1.24)	0.2840	0.0206
PhMA	0.54(0.26,1.13)	0.75(0.38,1.46)	0.5853	0.0412
2HPMA	0.55(0.26,1.17)	1.30(0.63,2.70)	0.3659	0.0294
MCaMA	0.97(0.48,1.93)	1.98(0.79,4.95)	0.1080	0.0118
34HBMA	1.01(0.46,2.20)	1.16(0.51,2.66)	0.7035	0.0500
4HBeMA	0.62(0.27,1.39)	1.36(0.51,3.65)	0.2495	0.0176
3HMPMA	0.66(0.31,1.43)	1.21(0.43,3.43)	0.5250	0.0382
4HMBeMA	1.17(0.55,2.47)	1.87(0.69,5.12)	0.2435	0.0147

Data are hazard ratios (95% confidence intervals), stratified by age groups, sex, residence, and smoking status, and adjusted for age (years), ethnicity, marital status, education, wealth score, body-mass index, nass use, physical activity, cotinine-creatinine ratio, pack-years of smoking, and history of previous cardiovascular events. The first tertiles were the reference categories. *P value of 0.05 with a false discovery rate control (i.e., 0.05×(the unadjusted p-value rank/the total number of biomarker comparisons)). †Significant associations with a false discovery rate control. Abbreviations are defined in Table 1

**Table 5:** Sensitivity analyses for the associations of volatile organic compound biomarkers and chronic respiratory disease mortality in the Golestan cohort study

	Based on adjusted models for creatinine* (149 cases and 545 non-cases)		Excluding the first 4 years of the follow-up (112 cases and 519 non-cases)	
	2nd vs. 1^St^ tertile	3rd vs. 1st tertile	2nd vs. 1^St^ tertile	3rd vs. 1st tertile
2MHA	1.52(0.76,3.05)	1.36(0.64,2.88)	0.87(0.37,2.04)	1.43(0.62,3.31)
3&4MHA	1.30(0.64,2.60)	1.08(0.51,2.31)	1.09(0.39,3.07)	1.39(0.58,3.32)
2CaEMA	1.21(0.52,2.83)	0.80(0.27,2.37)	0.79(0.34,1.85)	0.75(0.26,2.12)
2CyEMA	1.36(0.59,3.16)	1.99(0.59,6.73)	1.11(0.49,2.51)	1.45(0.45,4.74)
2HEMA	0.54(0.25,1.17)	0.64(0.28,1.46)	0.41(0.18,0.95)	0.44(0.17,1.10)
2CoEMA	1.43(0.61,3.33)	3.96(1.34,11.67)	1.02(0.39,2.68)	2.85(1.16,7.01)
3HPMA	1.04(0.41,2.65)	3.58(1.12,11.50)	0.91(0.37,2.22)	2.27(0.56,9.16)
BzMA	0.70(0.33,1.47)	1.28(0.57,2.88)	0.92(0.40,2.10)	0.94(0.41,2.14)
MADA	1.72(0.69,4.27)	5.97(1.99,17.92)	1.61(0.60,4.33)	3.82(1.29,11.29)
PhGA	0.36(0.17,0.78)	0.47(0.18,1.23)	0.40(0.15,1.01)	0.59(0.28,1.24)
PhMA	0.51(0.23,1.10)	0.90(0.39,2.08)	0.51(0.21,1.22)	0.50(0.23,1.12)
2HPMA	0.93(0.43,2.04)	1.42(0.60,3.35)	0.48(0.19,1.22)	1.17(0.51,2.68)
MCaMA	1.45(0.65,3.26)	1.76(0.66,4.72)	1.12(0.50,2.51)	2.20(0.85,5.72)
34HBMA	0.89(0.38,2.13)	0.87(0.25,2.99)	0.77(0.29,2.06)	1.11(0.42,2.89)
4HBeMA	0.65(0.25,1.65)	2.33(0.70,7.74)	0.63(0.25,1.58)	1.08(0.37,3.12)
3HMPMA	0.96(0.41,2.25)	1.95(0.61,6.17)	0.62(0.26,1.49)	0.97(0.31,2.99)
4HMBeMA	1.11(0.42,2.89)	2.55(0.65,9.95)	1.26(0.53,2.99)	1.77(0.57,5.56)

Data are hazard ratios (95% confidence intervals), stratified by age groups, sex, residence, and smoking status, and adjusted for age (years), ethnicity, marital status, education, wealth score, body-mass index, nass use, physical activity, cotinine-creatinine ratio, pack-years of smoking, and history of previous cardiovascular events. The first tertiles were the reference categories. *Using non-creatinine-corrected biomarkers in the models with creatinine as an independent covariate. Abbreviations are defined in Table 1

## Discussion

In this population-based case-cohort analysis, we found that biomarkers of acrolein and styrene/ethylbenzene were strongly associated with mortality from CRDs. These associations were independent of tobacco smoking intensity and duration, indicating the importance of non-tobacco sources of these VOCs in CRDs. Our findings persisted after excluding the first four years of the follow-up to show the stability of associations over time.

A previous history of CRDs may affect the concentrations of urinary VOC biomarkers. Because inhalation is the main route of VOC exposure, the absorption of some VOCs might be limited during the more advanced stages of disease progression. Either pathologic changes of respiratory system or modification of the patients’ behaviors during the chronic course of the disease may play a role in this observation. This can lead to reverse causation, showing biased “protective” associations between VOC biomarkers and respiratory diseases. The bias particularly poses as a limitation for cross-sectional and case-control studies assessing inhaled toxicants and respiratory diseases. For example, in a case-control study of the association between urinary VOC biomarkers and childhood asthma, the concentration of a styrene/ethylbenzene metabolite in asthmatic patients was significantly lower than healthy controls, and in the final regression model, after adjustment for age, gender, and BMI, the metabolite showed a protective association with the outcome [20]. Likewise, in a cross-sectional study to evaluate VOC exposures and the risk of chronic obstructive pulmonary disease in a random sample of the U.S. population, the blood concentrations of several VOCs tended to be lower in cases than healthy controls [21]. While reverse causality is an important limitation of these study designs, in our prospective cohort, we addressed this limitation by excluding individuals with a history of CRD and through sensitivity analyses of starting follow up 4 years after the baseline to further assess any potential bias.

Acrolein is a reactive unsaturated aldehyde with negative impacts on health (a probable human carcinogen) [22]. In the general population, the most important sources of acrolein exposure are tobacco smoke and overheated cooking oils. Biomass combustion and vehicle exhaust are other important sources [22]. Many studies in animal models have shown that acrolein is a serious threat to the respiratory system, however, limited epidemiologic studies are available about its respiratory effect [23]. Previous theoretical methods to provide a hazard prioritization for chemical constituents of cigarette smoke indicated that for respiratory diseases, acrolein outweighed all other constituents [24, 25]. Our results showed that acrolein biomarkers, especially 2CoEMA, were associated with subsequent CRD mortality in the general population. This association persisted after excluding the first four years of follow-up. In line with our results, some studies showed the association of urinary acrolein metabolites with a reduced lung function [11, 26] or respiratory disease [20]. In a study, forced expiratory volume in the first second (FEV1)% predicted, an indicator of the severity of airflow obstruction, was associated with urinary metabolites of acrolein in the U.S. adults [11]. A Chinese population-based adult cohort indicated an inverse relationship between acrolein exposure and pulmonary function tests cross-sectionally and during 3 years of follow-up. This relationship was aggravated/mediated by oxidative DNA damage, inflammation, and pulmonary epithelium injury that are associated with the onset and development of pulmonary diseases [26]. 

The styrene/ethylbenzene metabolite, MADA, in our study, had a strong and consistent association with CRD mortality, which was observed even after excluding the first four years of follow-up. Styrene is a high production volume chemical used in the manufacturing of reinforced plastics and synthetic rubbers [27, 28]. Ethylbenzene is almost exclusively used as an intermediate for styrene production [29]. The main routes of styrene exposure in the general population are inhalation of ambient air and cigarette smoke. Exposure by ingestion, e.g., microplastics in the aquatic environment or food packaging materials, may also significantly contribute to total exposure [27, 28]. Exposure to styrene is suspected to be related to adverse health outcomes, particularly neurologic disorders and cancers, and IARC has classified styrene as a probable human carcinogen [30]. However, according to the available evidence, chronic respiratory toxicity is not considered a known major concern for styrene exposure in human studies [7, 27]. Results of occupational studies about CRDs induced by styrene exposure are not conclusive [31]. For example, a study of boat-building workers reported a twofold increase in CRD mortality related to potentially high levels of environmental styrene [32]. However, a cohort study of 22,785 synthetic rubber industry employees (1943–2009) found that environmental styrene exposure was not related to non-malignant respiratory disease mortality [33]. A study showed that the healthy worker survivor bias—healthier workers stay employed longer—can strongly affect the results of styrene exposure health consequences in occupational cohorts [34]. Some studies from the National Health and Nutrition Examination Survey (NHANES), among the U.S. general population, evaluated the association of VOC exposures with spirometry-measured pulmonary function tests (i.e., FEV1 to forced vital capacity (FVC) ratio and FEV1% predicted).^10,11,35^ Among them, two studies found reduced pulmonary functions associated with styrene exposure [11, 35]. However, the changes in pulmonary function tests observed in these cross-sectional studies may have been transient or reversible [35]. Our longitudinal study, for the first time, found an association between the urinary metabolite of styrene and CRD mortality in the general population.

Some strengths of this study included using data from a general population-based cohort with a high participation rate and long-term follow-up, excluding participants with chronic respiratory diseases at baseline and in sensitivity analyses, highly reliable urinary VOC measurements by the CDC with rigorous quality control and quality assurance procedures, the hard outcome confirmed by a team of expert physicians, and the availability of several important covariates to minimize residual confounding. In this study design, all cases were included in 16 strata, while subcohort participants were randomly selected for each stratum. Weighted hazard ratios (HRs) were calculated as if the entire cohort had been used. This is an efficient way to reduce biomarker measurement costs while preserving the generalizability to the entire cohort and the temporal relationship between the exposure and the outcome [36]. Although tobacco smoking is the predominant risk factor for death due to CRDs, the association of an individual VOC with an outcome can be strongly affected by many other constituents in tobacco smoke. In this study to account for these constituents we stratified by smoking status and adjusted for urinary cotinine and pack-years of smoking. Cotinine is a specific nicotine metabolite and a well- known quantitative indicator of recent tobacco consumption. Urinary cotinine is a sensitive medium for the detection of even low-level exposure to tobacco smoke (e.g., second hand) as well as other nicotine-containing products [37]. 

Nevertheless, our study has some limitations. Urinary biomarkers were only measured in a single-spot urine sample, so potential changes in environmental sources and within individual variability in the uptake and metabolism of VOCs during the follow-up can bias our associations. However, using data of two urine sample taken 5 years apart from the same GCS participants, we previously found that many tobacco VOC biomarkers could be useful for exposure assessment in longitudinal studies, especially in the presence of strong sources of exposures such as tobacco smoking. Data of passive smoking were not collected in the GCS; however, the models were adjusted for urinary cotinine levels to diminish this limitation. The relatively small number of cases was another limitation that prevented us from conducting subgroup analysis. Although sources of parent VOCs may be different between the GCS and other populations; it cannot affect the direct associations observed between the urinary VOC biomarker concentrations and the outcome. Thus, the associations are applicable to other populations that are exposed to these compounds. Furthermore, we previously showed that the concentrations of VOC biomarkers were almost similar between general populations of the U.S. adult and GCS.

We found strong evidence for the role of VOC exposure in CRD mortality. Our study showed that the urinary metabolites of acrolein and styrene/ethylbenzene, measured in samples collected many years before the outcome, were associated with respiratory death among the general population independent of tobacco smoking. These results highlight the importance of controlling the exposure to the main sources of acrolein (burning organic materials) and styrene/ethylbenzene (i.e., plastics and synthetic rubbers), in addition to tobacco smoking.

## Electronic supplementary material

Below is the link to the electronic supplementary material.

Supplementary Material 1

## Data Availability

No datasets were generated or analysed during the current study.

## Declarations

### Disclaimer

The findings and conclusions in this report are those of the authors and do not necessarily represent the official position of the U.S. Department of Health and Human Services or any of its affiliated institutions or agencies. Use of trade names and commercial sources is for identification only and does not constitute endorsement by the U.S. Department of Health and Human Services, or the U.S. Centers for Disease Control and Prevention (Division of Laboratory Sciences).

## References

[1] Chronic respiratory diseases. World Health Organization. 2023. [Available at: https://www.who.int/health-topics/chronic-respiratory-diseases#tab=tab_1, accessed: 8 December 2023].

[2] Global burden of chronic respiratory diseases and risk factors. 1990–2019: an update from the global burden of disease study 2019. EClinicalMedicine. 2023;59:101936. 37229504 10.1016/j.eclinm.2023.101936 PMC7614570

[3] Stolz D, Mkorombindo T, Schumann DM, Agusti A, Ash SY, Bafadhel M, et al. Towards the elimination of chronic obstructive pulmonary disease: a lancet commission. Lancet. 2022;400(10356):921–72. 36075255 10.1016/S0140-6736(22)01273-9 PMC11260396

[4] Williams S, Sheikh A, Campbell H, Fitch N, Griffiths C, Heyderman RS, et al. Respiratory research funding is inadequate, inequitable, and a missed opportunity. Lancet Respir Med. 2020;8(8):e67–8. 32763207 10.1016/S2213-2600(20)30329-5 PMC7402663

[5] McDonald BC, de Gouw JA, Gilman JB, Jathar SH, Akherati A, Cappa CD, et al. Volatile chemical products emerging as largest petrochemical source of urban organic emissions. Science. 2018;359(6377):760–4. 29449485 10.1126/science.aaq0524

[6] Alwis KU, Blount BC, Britt AS, Patel D, Ashley DL. Simultaneous analysis of 28 urinary VOC metabolites using ultra high performance liquid chromatography coupled with electrospray ionization tandem mass spectrometry (UPLC-ESI/MSMS). Anal Chim Acta. 2012;750:152–60. 23062436 10.1016/j.aca.2012.04.009 PMC11261307

[7] U.S. Food and Drug Administration. Harmful and Potentially Harmful Constituents in Tobacco Products and Tobacco Smoke: Established List 2012 [Available from: https://www.fda.gov/tobacco-products/rules-regulations-and-guidance/harmful-and-potentially-harmful-constituents-tobacco-products-and-tobacco-smoke-established-list, accessed: 8 December 2024.].

[8] Liu N, Bu Z, Liu W, Kan H, Zhao Z, Deng F, et al. Health effects of exposure to indoor volatile organic compounds from 1980 to 2017: A systematic review and meta-analysis. Indoor Air. 2022;32(5):e13038. 35622720 10.1111/ina.13038

[9] McNamee R. Confounding and confounders. Occup Environ Med. 2003;60(3):227–34. quiz 164, 234. 12598677 10.1136/oem.60.3.227 PMC1740493

[10] Elliott L, Longnecker MP, Kissling GE, London SJ. Volatile organic compounds and pulmonary function in the third National health and nutrition examination survey, 1988–1994. Environ Health Perspect. 2006;114(8):1210–4. 16882527 10.1289/ehp.9019 PMC1551996

[11] Mendy A, Burcham S, Merianos AL, Mersha TB, Mahabee-Gittens EM, Chen A, Yolton K. Urinary volatile organic compound metabolites and reduced lung function in U.S. Adults. Respir Med. 2022;205:107053. 36399896 10.1016/j.rmed.2022.107053 PMC9869342

[12] Etemadi A, Abnet CC, Dawsey SM, Freedman ND. Biomarkers of tobacco carcinogenesis in diverse populations: challenges and opportunities. Cancer Epidemiol Biomarkers Prev. 2023;32(3):289–91. 36872902 10.1158/1055-9965.EPI-22-1289

[13] Pourshams A, Khademi H, Malekshah AF, Islami F, Nouraei M, Sadjadi AR, et al. Cohort profile: the Golestan cohort Study–a prospective study of oesophageal cancer in Northern Iran. Int J Epidemiol. 2010;39(1):52–9. 19332502 10.1093/ije/dyp161 PMC3709199

[14] Bernert JT, Harmon TL, Sosnoff CS, McGuffey JE. Use of continine immunoassay test strips for preclassifying urine samples from smokers and nonsmokers prior to analysis by LC-MS-MS. J Anal Toxicol. 2005;29(8):814–8. 16374940 10.1093/jat/29.8.814

[15] Etemadi A, Poustchi H, Chang CM, Blount BC, Calafat AM, Wang L, et al. Urinary biomarkers of carcinogenic exposure among cigarette, waterpipe, and smokeless tobacco users and never users of tobacco in the Golestan cohort study. Cancer Epidemiol Biomarkers Prev. 2019;28(2):337–47. 30622099 10.1158/1055-9965.EPI-18-0743 PMC6935158

[16] Islami F, Kamangar F, Nasrollahzadeh D, Aghcheli K, Sotoudeh M, Abedi-Ardekani B, et al. Socio-economic status and oesophageal cancer: results from a population-based case-control study in a high-risk area. Int J Epidemiol. 2009;38(4):978–88. 19416955 10.1093/ije/dyp195 PMC2720396

[17] Khademi H, Etemadi A, Kamangar F, Nouraie M, Shakeri R, Abaie B, et al. Verbal autopsy: reliability and validity estimates for causes of death in the Golestan cohort study in Iran. PLoS ONE. 2010;5(6):e11183. 20567597 10.1371/journal.pone.0011183 PMC2887437

[18] Barlow WE, Ichikawa L, Rosner D, Izumi S. Analysis of case-cohort designs. J Clin Epidemiol. 1999;52(12):1165–72. 10580779 10.1016/s0895-4356(99)00102-x

[19] Benjamini Y, Hochberg Y. Controlling the false discovery rate: A practical and powerful approach to multiple testing. J R Stat Soc Ser B. 1995;57:289–300.

[20] Kuang H, Li Z, Lv X, Wu P, Tan J, Wu Q, et al. Exposure to volatile organic compounds May be associated with oxidative DNA damage-mediated childhood asthma. Ecotoxicol Environ Saf. 2021;210:111864. 33412282 10.1016/j.ecoenv.2020.111864

[21] Lv JJ, Li XY, Shen YC, You JX, Wen MZ, Wang JB, Yang XT. Assessing volatile organic compounds exposure and chronic obstructive pulmonary diseases in US adults. Front Public Health. 2023;11:1210136. 37475768 10.3389/fpubh.2023.1210136 PMC10354632

[22] International Agency for Research on Cancer. Acrolein, Crotonaldehyde, and Arecoline. IARC monographs on the evaluation of carcinogenic risks to humans. Volume 128. Lyon, FR: IARC; 2021. 36924508

[23] U.S. Department of Health and Human Services. Public Health Service Agency for Toxic Substances and Disease Registry. Toxicological profile for acrolein 2007 (Available at: https://www.atsdr.cdc.gov/toxprofiles/tp124.pdf, accessed: 8 December 2023).

[24] Haussmann HJ. Use of hazard indices for a theoretical evaluation of cigarette smoke composition. Chem Res Toxicol. 2012;25(4):794–810. 22352345 10.1021/tx200536w

[25] Fowles J, Dybing E. Application of toxicological risk assessment principles to the chemical constituents of cigarette smoke. Tob Control. 2003;12(4):424–30. 14660781 10.1136/tc.12.4.424 PMC1747794

[26] Wang B, Yu L, Liu W, Yang M, Fan L, Zhou M, et al. Cross-sectional and longitudinal associations of acrolein exposure with pulmonary function alteration: assessing the potential roles of oxidative DNA damage, inflammation, and pulmonary epithelium injury in a general adult population. Environ Int. 2022;167:107401. 35850081 10.1016/j.envint.2022.107401

[27] Banton MI, Bus JS, Collins JJ, Delzell E, Gelbke HP, Kester JE, et al. Evaluation of potential health effects associated with occupational and environmental exposure to styrene - an update. J Toxicol Environ Health B Crit Rev. 2019;22(1–4):1–130. 31284836 10.1080/10937404.2019.1633718

[28] Yuan Z, Nag R, Cummins E. Human health concerns regarding microplastics in the aquatic environment - From marine to food systems. Sci Total Environ. 2022;823:153730. 35143789 10.1016/j.scitotenv.2022.153730

[29] International Agency for Research on Cancer. Some industrial chemicals. IARC monographs on the evaluation of carcinogenic risks to humans. Volume 77. Lyon, FR: IARC; 2000. pp. 227–66.

[30] International Agency for Research on Cancer. Styrene, styrene-7,8-oxide, and Quinoline. IARC monographs on the evaluation of carcinogenic risks to humans. Volume 121. Lyon, FR: IARC; 2019. pp. 32–5. 31967769

[31] Nett RJ, Cox-Ganser JM, Hubbs AF, Ruder AM, Cummings KJ, Huang YT, Kreiss K. Non-malignant respiratory disease among workers in industries using styrene-A review of the evidence. Am J Ind Med. 2017;60(2):163–80. 28079275 10.1002/ajim.22655 PMC5379121

[32] Ruder AM, Meyers AR, Bertke SJ. Mortality among styrene-exposed workers in the reinforced plastic boatbuilding industry. Occup Environ Med. 2016;73(2):97–102. 26574575 10.1136/oemed-2015-102990

[33] Sathiakumar N, Tipre M, Leader M, Brill I, Delzell E. Mortality among men and women in the North American synthetic rubber industry, 1943 to 2009. J Occup Environ Med. 2019;61(11):887–97. 31464816 10.1097/JOM.0000000000001688

[34] Bertke SJ, Keil AP, Daniels RD. Lung Cancer mortality and styrene exposure in the Reinforced-Plastics boatbuilding industry: evaluation of healthy worker survivor Bias. Am J Epidemiol. 2021;190(9):1784–92. 33847736 10.1093/aje/kwab108 PMC8628256

[35] Mendy A, Merianos AL, Mersha TB, Mahabee-Gittens EM. Blood volatile organic compounds associated with non-reversible and reversible airflow obstruction in US adults. Eur Respir J. 2022;60(5). 10.1183/13993003.01185-2022 PMC10191183 36202413

[36] O’Brien KM, Lawrence KG, Keil AP. The case for case-Cohort: an applied epidemiologist’s guide to reframing case-Cohort studies to improve usability and flexibility. Epidemiology. 2022;33(3):354–61. 35383643 10.1097/EDE.0000000000001469 PMC9172927

[37] Yuan JM, Butler LM, Stepanov I, Hecht SS. Urinary tobacco smoke-constituent biomarkers for assessing risk of lung cancer. Cancer Res. 2014;74(2):401–11. 24408916 10.1158/0008-5472.CAN-13-3178 PMC4066207

