# Supplementary material for: Exposure to volatile organic compounds and chronic respiratory disease mortality, a case-cohort study
Source: Respir Res. 2025 Mar 5;26:88. doi: 10.1186/s12931-025-03165-1 (PMC11884121; doi:10.1186/s12931-025-03165-1)
Supplement: Supplementary file 1 — Supplementary Material 1 [file 12931_2025_3165_MOESM1_ESM.docx]

**Supplement**

**Table S1.** Selection of cases and the stratified sub-cohort with sampling fractions^*^

|  | Current smoking | | | Non-current smoking | | |
| --- | --- | --- | --- | --- | --- | --- |
|  | Stratum number† | Case | Random sample from all in the stratum | Stratum number† | Case | Random sample from all in the stratum |
| Urban male |  |  |  |  |  |  |
| <55 years | 1 | 4 | 14 (14/746=.019) | 9 | 2 | 11 (11/1502=.0073) |
| ≥55 years | 2 | 12 | 36 (36/349=.103) | 10 | 13 | 36 (36/1148=.0314) |
| Rural male |  |  |  |  |  |  |
| <55 years | 3 | 15 | 60 (60/2926=.021) | 11 | 21 | 51 (51/7626=.0067) |
| ≥55 years | 4 | 27 | 73 (73/1005=.073) | 12 | 53 | 101 (101/4626=.0218) |
| Urban female |  |  |  |  |  |  |
| <55 years | 5 | 0 | 2 (2/74=.027) | 13 | 6 | 15 (15/3775=.0040) |
| ≥55 years | 6 | 0 | 4 (4/68=.059) | 14 | 7 | 34 (34/1943=.0175) |
| Rural female |  |  |  |  |  |  |
| <55 years | 7 | 0 | 5 (5/239=.021) | 15 | 28 | 50 (50/14598=.0034) |
| ≥55 years | 8 | 4 | 11 (11/134=.082) | 16 | 50 | 107 (107/6396=.0167) |
| Totals  (included/eligible) |  | 62/62 | 205/5541 |  | 180/180 | 405/41614 |

*Sampling fraction=the number of included individuals in each stratum/ total individuals in the stratum with a urine sample available. †All participants were stratified into 16 strata. Each stratum contained all cases and a random sample of subcohort participants in that stratum.

**Table S2**: Baseline characteristics of the subcohort and all cohort participants

| Variables | | Subcohort  (n=610) | All cohort  (n=50045) |
| --- | --- | --- | --- |
|  |  |  |  |
| Age (years) | | 57.8 (9.4) | 52.06 (8.9) |
| Sex, women | | 228 (37.4) | 28811 (57.6) |
| Residence, rural | | 458 (75.1) | 40011 (80) |
| Current smoking | | 204 (33.4) | 5449 (10.9) |
| Ethnicity, Turkmen | | 421 (69.0) | 37253 (74.4) |
| Marital status, married | | 520 (85.3) | 43955 (87.8) |
| Previous cardiovascular events | | 54 (8.9) | 3371 (6.7) |
| Body-mass index (kg/m^2^) | <18.5 | 57 (9.3) | 2410 (4.8) |
|  | 18.5-24.9 | 264 (43.2) | 17965 (35.9) |
|  | 25-29.9 | 174 (28.5) | 16937 (33.9) |
|  | ≥30 | 115 (18.9) | 12725 (25.4) |
| Education | No | 429 (70.3) | 35118 (70.2) |
|  | ≤5 years | 98 (16.1) | 8463 (16.9) |
|  | >5 years | 83 (13.6) | 6464 (12.9) |
| Wealth score | 1^st^ terile | 250 (41.0) | 17906 (35.8) |
|  | 2^nd^ tertile | 169 (27.7) | 15471 (30.9) |
|  | 3^rd^ tertile | 191 (31.3) | 16668 (33.3) |
| Physical activity | 1^st^ terile | 213 (34.9) | 16582 (33.1) |
|  | 2^nd^ tertile | 171 (28.0) | 15439 (30.9) |
|  | 3^rd^ tertile | 199 (32.6) | 15928 (31.8) |
|  | unknown | 27 (4.4) | 2096 (4.2) |
| Nass use | Never users | 536 (87.9) | 46167 (92.3) |
|  | Past users | 16 (2.6) | 704 (1.4) |
|  | Current users | 58 (9.5) | 3174 (6.3) |

Data are mean (SD) or n (%).

**Table S3.** Analytical limit of detection of volatile organic compound biomarkers in urine

| Acronym | LOD (ng/ml) | N (%)<LOD^*^ | Coefficient of variation (CV) |
| --- | --- | --- | --- |
| 2MHA | 5 | 8 (1.15) | 13% |
| 3&4MHA | 8 | 5 (0.72) | 8% |
| 2CaEMA | 2.2 | 6 (0.86) | 9% |
| 2CaHEMA | 9.4 | 398 (57.43)^†^ | 14% |
| 1CyHEMA | 2.6 | 437 (62.97) | 5% |
| 2CyEMA | 0.5 | 90 (12.97) | 13% |
| 2HEMA | 0.791 | 164 (23.63) | 13% |
| 2CoEMA | 6.96 | 13 (1.87) | 7% |
| 3HPMA | 13 | 17 (2.45) | 10% |
| BzMA | 0.5 | 22 (3.17) | 6% |
| MADA | 12 | 5 (0.72)^†^ | 9% |
| PhGA | 12 | 82 (11.82) | 14% |
| PhMA | 0.6 | 210 (30.26) | 13% |
| 2HPMA | 5.3 | 50 (7.20) | 14% |
| MCaMA | 6.26 | 12 (1.73) | 7% |
| 34HBMA | 5.25 | 4 (0.58) | 6% |
| 4HBeMA | 0.6 | 23 (3.31) | 11% |
| 3HMPMA | 1.695 | 3 (0.43) | 7% |
| 4HMBeMA | 1.2 | 118 (17.00) | 17% |
| TTCA | 11.2 | 574 (82.71) | 8% |

LOD denotes limit of detection. *N (%) of urine samples with biomarkers below the LOD. †There were one missing value for 2CaHEMA and one for MADA concentrations. Abbreviations are defined in Table 1.

**Table S4.** Geometric means of biomarkers in cases with and without previous history of chronic respiratory diseases (CRD) at baseline cohort

|  | Without baseline CRDs  (n=149) | With baseline CRDs  (n=92) | p |
| --- | --- | --- | --- |
| 2MHA | 112.48 (90.70,139.49) | 91.44 (70.86,118.00) | 0.228 |
| 3&4MHA | 499.88 (407.61,613.04) | 390.08 (310.78,489.61) | 0.121 |
| 2CaEMA | 79.94 (65.85,97.04) | 70.50 (57.65,86.20) | 0.397 |
| 2CaHEMA | 9.30 (7.58,11.41) | 8.79 (6.68,11.57) | 0.741 |
| 1CyHEMA | 4.03 (2.91,5.59) | 2.25 (1.59,3.19) | 0.022 |
| 2CyEMA | 9.50 (6.48,13.93) | 5.01 (3.34,7.53) | 0.031 |
| 2HEMA | 1.88 (1.54,2.29) | 1.46 (1.15,1.87) | 0.123 |
| 2CoEMA | 134.20 (115.96,155.31) | 101.02 (85.42,119.47) | 0.014 |
| 3HPMA | 391.88 (314.55,488.23) | 296.61 (230.13,382.30) | 0.110 |
| BzMA | 6.50 (5.43,7.78) | 6.57 (5.50,7.85) | 0.939 |
| MADA | 335.14 (298.69,376.03) | 285.21 (252.28,322.43) | 0.070 |
| PhGA | 77.48 (61.66,97.37) | 88.15 (67.30,115.45) | 0.479 |
| PhMA | 1.09 (0.91,1.30) | 0.99 (0.81,1.21) | 0.493 |
| 2HPMA | 30.04 (25.58,35.29) | 28.71 (24.36,33.84) | 0.712 |
| MCaMA | 230.76 (193.59,275.07) | 181.83 (146.27,226.04) | 0.095 |
| 34HBMA | 351.15 (312.46,394.63) | 326.42 (288.19,369.72) | 0.417 |
| 4HBeMA | 9.52 (7.51,12.06) | 7.04 (5.58,8.88) | 0.091 |
| 3HMPMA | 377.51 (307.52,463.42) | 294.45 (239.47,362.04) | 0.111 |
| 4HMBeMA | 6.29 (4.76,8.31) | 4.49 (3.52,5.72) | 0.098 |
| TTCA | 8.01 (6.40,10.03) | 7.80 (5.56,10.94) | 0.892 |

Abbreviations are defined in Table 1.

**Table S5**. Weighted geometric means and 95% confidence intervals of volatile organic compound biomarkers (µg/g creatinine), based on smoking status.

|  | Smoking (n=230) | Non-smoking (n=464) | p |
| --- | --- | --- | --- |
| 2MHA | 168.23 (147.53,191.84) | 89.04 (79.56,99.65) | <0.0001 |
| 3&4MHA | 825.84 (731.93,931.79) | 341.51 (306.13,380.98) | <0.0001 |
| 2CaEMA | 145.54 (130.61,162.17) | 56.28 (51.66,61.31) | <0.0001 |
| 2CaHEMA | 14.85 (12.90,17.09) | 6.96 (6.22,7.80) | <0.0001 |
| 1CyHEMA | 18.99 (15.80,22.82) | 1.50 (1.31,1.72) | <0.0001 |
| 2CyEMA | 80.19 (66.53,96.64) | 1.98 (1.70,2.30) | <0.0001 |
| 2HEMA | 3.54 (3.04,4.12) | 1.41 (1.27,1.56) | <0.0001 |
| 2CoEMA | 195.23 (175.93,216.63) | 89.29 (83.44,95.56) | <0.0001 |
| 3HPMA | 962.77 (849.61,1091.00) | 215.68 (198.96,233.80) | <0.0001 |
| BzMA | 6.30 (5.67,6.99) | 5.64 (5.14,6.19) | 0.152 |
| MADA | 409.62 (379.10,442.61) | 223.73 (211.21,237.00) | <0.0001 |
| PhGA | 103.42 (87.48,122.28) | 85.45 (76.89,94.95) | 0.049 |
| PhMA | 1.39 (1.24,1.57) | 0.90 (0.81,0.99) | <0.0001 |
| 2HPMA | 51.31 (46.13,57.08) | 24.27 (22.38,26.31) | <0.0001 |
| MCaMA | 424.64 (380.19,474.30) | 126.43 (116.10,137.68) | <0.0001 |
| 34HBMA | 420.01 (399.08,442.04) | 307.82 (289.34,327.47) | <0.0001 |
| 4HBeMA | 26.35 (23.28,29.81) | 5.34 (4.90,5.82) | <0.0001 |
| 3HMPMA | 900.77 (800.33,1013.82) | 224.80 (206.60,244.61) | <0.0001 |
| 4HMBeMA | 25.32 (21.27,30.12) | 2.76 (2.52,3.03) | <0.0001 |
| TTCA | 7.49 (6.27,8.94) | 6.95 (6.05,7.99) | 0.535 |

Abbreviations are defined in Table 1.

**Table S6.** Correlations of creatinine-corrected biomarker concentrations in the sub-cohort population*

|  | 2MHA | 3&4MHA | 2CaEMA | 2CaHEMA | 1CyHEMA | 2CyEMA | 2HEMA | 2CoEMA | 3HPMA | BzMA | MADA | PhGA | PhMA | 2HPMA | MCaMA | 34HBMA | 4HBeMA | 3HMPMA | 4HMBeMA | TTCA |
| --- | --- | --- | --- | --- | --- | --- | --- | --- | --- | --- | --- | --- | --- | --- | --- | --- | --- | --- | --- | --- |
| 2MHA | 1 |  |  |  |  |  |  |  |  |  |  |  |  |  |  |  |  |  |  |  |
| 3&4MHA | .91 | 1 |  |  |  |  |  |  |  |  |  |  |  |  |  |  |  |  |  |  |
| 2CaEMA | .18 | .29 | 1 |  |  |  |  |  |  |  |  |  |  |  |  |  |  |  |  |  |
| 2CaHEMA | .16 | .24 | .84 | 1 |  |  |  |  |  |  |  |  |  |  |  |  |  |  |  |  |
| 1CyHEMA | .24 | .38 | .53 | .48 | 1 |  |  |  |  |  |  |  |  |  |  |  |  |  |  |  |
| 2CyEMA | .25 | .4 | .57 | .49 | .93 | 1 |  |  |  |  |  |  |  |  |  |  |  |  |  |  |
| 2HEMA | .12 | .19 | .27 | .26 | .53 | .51 | 1 |  |  |  |  |  |  |  |  |  |  |  |  |  |
| 2CoEMA | .28 | .38 | .41 | .36 | .68 | .73 | .46 | 1 |  |  |  |  |  |  |  |  |  |  |  |  |
| 3HPMA | .26 | .4 | .51 | .4 | .78 | .86 | .51 | .75 | 1 |  |  |  |  |  |  |  |  |  |  |  |
| BzMA | -.08 | -.06 | .16 | .15 | .01 | .02 | .02 | .07 | .03 | 1 |  |  |  |  |  |  |  |  |  |  |
| MADA | .59 | .66 | .49 | .41 | .66 | .69 | .36 | .53 | .59 | .16 | 1 |  |  |  |  |  |  |  |  |  |
| PhGA | .19 | .24 | .16 | .12 | .13 | .14 | .04 | .26 | .25 | .18 | .25 | 1 |  |  |  |  |  |  |  |  |
| PhMA | .29 | .24 | .15 | .12 | .26 | .23 | .15 | .2 | .23 | -.02 | .4 | .11 | 1 |  |  |  |  |  |  |  |
| 2HPMA | .21 | .26 | .26 | .21 | .48 | .46 | .34 | .44 | .47 | .03 | .41 | .08 | .27 | 1 |  |  |  |  |  |  |
| MCaMA | .21 | .38 | .76 | .67 | .75 | .78 | .39 | .56 | .68 | .11 | .64 | .16 | .21 | .38 | 1 |  |  |  |  |  |
| 34HBMA | .15 | .26 | .44 | .33 | .45 | .48 | .3 | .59 | .59 | .26 | .43 | .35 | .25 | .37 | .45 | 1 |  |  |  |  |
| 4HBeMA | .26 | .41 | .43 | .34 | .8 | .86 | .48 | .74 | .86 | .01 | .58 | .2 | .22 | .52 | .64 | .52 | 1 |  |  |  |
| 3HMPMA | .24 | .39 | .42 | .32 | .76 | .83 | .5 | .72 | .9 | .03 | .56 | .22 | .21 | .53 | .63 | .55 | .93 | 1 |  |  |
| 4HMBeMA | .2 | .36 | .4 | .3 | .79 | .83 | .5 | .66 | .84 | <.01 | .53 | .14 | .17 | .43 | .64 | .43 | .92 | .88 | 1 |  |
| TTCA | -.04 | -.04 | .04 | .01 | -.01 | <.01 | <.01 | .05 | .03 | .07 | -.02 | .01 | <.01 | .02 | <.01 | .05 | .03 | .02 | .03 | 1 |

*After excluding participants with previous history of chronic respiratory diseases.
